# Delirium in hospitalized COVID-19 patients: Predictors and implications for patient outcome

**DOI:** 10.1371/journal.pone.0278214

**Published:** 2022-12-22

**Authors:** Vera Wilke, Mihaly Sulyok, Maria-Ioanna Stefanou, Vivien Richter, Benjamin Bender, Ulrike Ernemann, Ulf Ziemann, Nisar Malek, Katharina Kienzle, Constantin Klein, Stefanie Bunk, Siri Goepel, Annerose Mengel

**Affiliations:** 1 Department of Neurology and Stroke, University Hospital Tuebingen, Tuebingen, Germany; 2 Hertie-Institute for Clinical Brain Research, University of Tuebingen, Tuebingen, Germany; 3 Department of Pathology and Neuropathology, Eberhard Karls University of Tuebingen, Tuebingen, Germany; 4 Second Department of Neurology, Medical School, National and Kapodistrian University of Athens, ’Attikon’ University General Hospital, Athens, Greece; 5 Department of Diagnostic and Interventional Neuroradiology, University Hospital Tuebingen, Tuebingen, Germany; 6 Department of Internal Medicine I, University Hospital of Tuebingen, Tuebingen, Germany; 7 Clinical Research Unit Tuebingen, German Center of Infectious Diseases (DZIF), Brunswick, Germany; Bolu Abant İzzet Baysal University: Bolu Abant Izzet Baysal Universitesi, TURKEY

## Abstract

**Introduction:**

Delirium is recognized as a severe complication of coronavirus-disease-2019 (COVID-19). COVID-19-associated delirium has been linked to worse patient outcomes and is considered to be of multifactorial origin. Here we sought to evaluate the incidence and risk factors of delirium in hospitalized COVID-19 patients, along with its impact on clinical outcome.

**Methods:**

Consecutive adult COVID-19 patients admitted to a tertiary academic referral hospital between March 1^st^ and December 31^st^, 2020 were included. Potential risk factors for delirium were evaluated, including: age, gender, disease severity (as per the highest WHO grading reached during admission), laboratory parameters for infection and renal function (as per their most extreme values), and presence of comorbidities. To assess the relative strength of risk factors for predicting the occurrence of delirium, we performed a random-forest survival analysis.

**Results:**

347 patients with positive COVID-19 PCR test and median age 68.2 [IQR 55.5, 80.5] years were included. Of those, 79 patients (22.8%) developed delirium, 81 (23.3%) were transferred to ICU, 58 (16.7%) died. 163 (73.8%) patients were discharged home, 13 (5.9%) to another hospital, 32 (14.5%) to nursing homes, 13 (5.9%) to rehabilitation with an overall median admission-to-discharge time of 53 [IQR 14, 195] days. The strongest predictors for the occurrence of delirium were blood urea nitrogen (minimal depth value (MD): 3.33), age (MD: 3.75), disease severity (as captured by WHO grading; MD: 3.93), leukocyte count (MD: 4.22), the presence of a neurodegenerative history (MD: 4.43), ferritin (MD: 4.46) and creatinine (MD: 4.59) levels.

**Conclusion:**

The risk of delirium in COVID-19 can be stratified based on COVID-19 disease severity and–similar to delirium associated with other respiratory infections–the factors advanced age, neurodegenerative disease history, and presence of elevated infection and renal-retention parameters. Screening for these risk factors may facilitate early identification of patients at high-risk for COVID-19-associated delirium.

## Introduction

Delirium is increasingly recognized as a severe neurological complication of coronavirus disease 2019 (COVID-19) [1,2]. Recent studies indicate that the relative incidence of delirium in COVID-19 amounts to 30% among all hospitalized patients and 80% among intensive care unit (ICU) patients [1,3,4].

COVID-19-associated delirium is currently considered to be of multifactorial origin [5], even though direct neurotropic effects of the severe acute respiratory syndrome coronavirus 2 (SARS-CoV-2) might also contribute to its pathogenesis [6,7]. Known risk factors for the occurrence of delirium in COVID-19 comprise well-established predisposing (e.g., age and cognitive deficits) [8] and precipitating factors of delirium (e.g., electrolyte disturbances and concomitant treatments) [9,10], but also COVID-19 related risk factors, including the severe respiratory distress caused by SARS-CoV-2 and the social distancing and patient isolation measures implemented during the COVID-19 pandemic era [11].

A better understanding of COVID-19-associated delirium appears warranted as its occurrence has been linked to worse patient outcomes, including longer treatment duration, higher complication rates, increased mortality [3,11–13] and, in COVID-19 survivors, consecutive cognitive decline [2,13,14]. As further infection waves remain likely and COVID-19 may serve as a paradigmatic model for acute respiratory distress syndromes, COVID-19-associated delirium requires further research, particularly in terms of its predisposing factors and its implications for patient outcome.

The goals of our retrospective study were (1) to assess the incidence of delirium in a large cohort of consecutive, hospitalized COVID-19 patients treated at a German tertiary referral center, (2) to identify predictors of the occurrence of delirium in hospitalized COVID-19 patients, by comprehensively screening demographic, clinical, laboratory and neuroradiological features, as well as capturing disease severity in a standardized manner, and (3) to scrutinize whether the occurrence of delirium in COVID-19 is indeed associated with worse patient outcomes in terms of treatment duration, discharge modality, and mortality.

## Materials and methods

### Study design and regulations

We retrospectively analyzed prospectively acquired data of hospitalized COVID-19 patients with the aim to assess the incidence, the risk factors, and the impact of delirium on clinical outcome. The ethics committee of the University of Tuebingen approved the study (Protocol number: 431/2020BO), hereby individual informed consent was waived due to the retrospective nature of the analysis of de-identified patient data.

### Subjects

Our study cohort included all consecutive adult COVID-19 patients treated at the University Hospital Tuebingen, a tertiary academic referral center, between March 1^st^ 2020 and December 31^st^ 2020. COVID-19 was diagnosed on the basis of the WHO diagnostic criteria for definite cases [15]. Patients were excluded if the duration of the hospital stay was less than 24 hours or if the patient was not assessed for the presence of delirium during the hospital stay (exclusion rate: 13.6%). We assessed the disease severity of COVID-19 according to previously published grading recommendations [16].

### Study assessments

The diagnosis of delirium was based on the Diagnostic and Statistical Manual of the American Psychiatric Association (DSM-5) criteria for non-ICU patients [17], and the Intensive Care Delirium Screening Checklist (ICDSC) for ICU patients [18]. To identify possible risk factors for the occurrence of delirium in COVID-19, we assessed the following factors: age, gender, disease severity as captured by the WHO grading scale, laboratory parameters for infection (leukocyte count, C-reactive protein (CRP), ferritin, interleukin-6 (IL-6), procalcitonin (PCT)) and renal function (creatinine, blood urea nitrogen (BUN)), and the presence of comorbidities. For the analysis of the laboratory measurements, the most extreme values recorded during the hospitalization were assessed (e.g., max. CRP level, min. oxygen saturation). Comorbidities were retrieved from the medical records.

A subgroup analysis was performed for a sub-cohort of neuroradiologically assessed patients. This sub-cohort comprised all COVID-19 in-patients who were assessed by cranial computed tomography (CT) and/or magnetic resonance imaging (MRI) within four weeks after their first positive COVID-19 PCR test. For these patients, structured neuroradiological reports were extracted from the hospital information system and images were re-assessed by an independent consultant neuroradiologist, blinded to the clinical data (39 patients with CT scans, 20 with MRI scans, including 7 patients with both CT and MRI).

### Primary endpoints

The primary endpoint of the study was the presence of delirium in our SARS-CoV-2 positive patients.

### Secondary endpoints

The secondary endpoints of the study were: (i) incidence and (ii) risk factors of delirium in hospitalized COVID-19 patients, (iii) the effects of delirium on clinical outcome.

### Statistical analysis

Group differences between COVID-19 patients with and without delirium were assessed using Fisher’s exact tests and two-tailed independent-sample Mann-Whitney U tests, depending on the data properties (i.e., categorical vs. continuous variables). Missing data were imputed using a random forest algorithm of the MissForest package as described by Stekhoven et al. (2012) (15). In the subgroup of neuroradiologically assessed patients, group differences between patients with and without delirium were explored accordingly. Data were reported as mean ± standard deviation (SD) for normally distributed data, median and interquartile range (IQR) for non-normally distributed data, and as absolute numbers/percentages for categorical variables. Holm-correction was performed to counteract multiple-testing problems.

### Random-forest modelling

To assess the relative importance of variables for predicting the occurrence of delirium, we performed a random-forest survival analysis using the RandomForestSRC package with log-rank “gini random” as splitting rule [19]. We randomly split the data into a training (80%) and a validation (20%) dataset. We grew the forests consisting of 1000 trees each with the training dataset, using sampling without replacement [20]. The random-forest modelling included all available variables. The relative importance of the variables was assessed by their minimal depth values, based on a permutation approach [21]. The validation dataset was used for validation purposes. All calculations were performed with R 4.0.1. The anonymized data and the analysis script of this article can be accessed on reasonable request addressed to the corresponding author if in accordance with the approval of the ethics committee.

## Results

### Cohort characteristics

**Fig 1:**
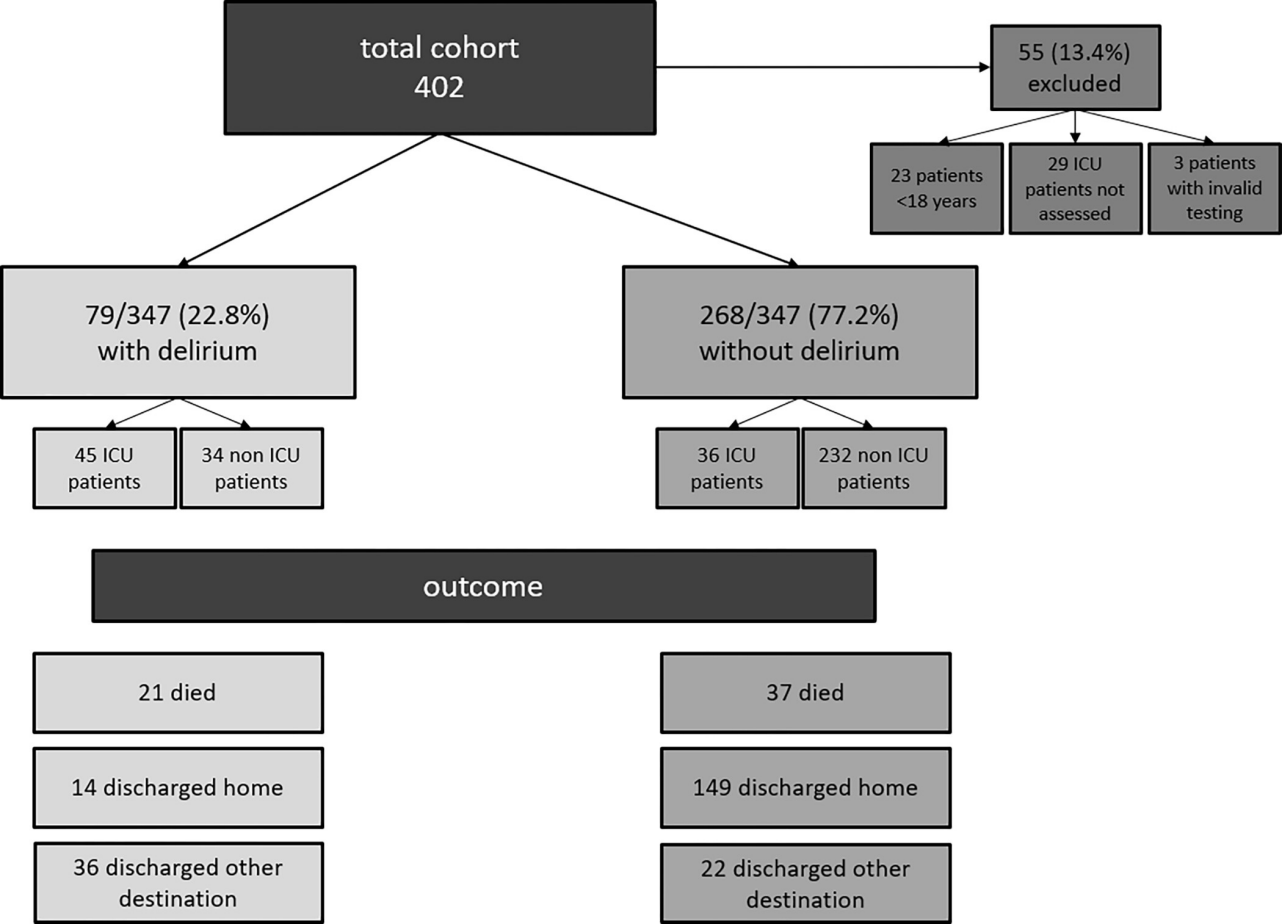
Incidence of delirium and mortality in the study cohort.

Altogether, our study cohort consisted of 347 individuals with a positive SARS-CoV-2 PCR test result. 79 (22.8%) of these individuals had delirium, 81 (23.3%) were transferred to ICU, and 58 (16.7%) died (Fig 1). The median time from admission to discharge was 53 days (IQR 14–195). Most patients were discharged home (163, 73.8%), the remaining patients were discharged to another hospital (13, 5.9%), to nursing homes (32, 14.5%) or to rehabilitation (13, 5.9%). The median age was 68.2 years (IQR 55.5–80.5). The proportion of women was 46%.

### Demographic, clinical and laboratory features associated with delirium

We analyzed the association of the occurrence of delirium with demographic characteristics (age and gender), clinical characteristics (disease severity as captured by the COVID-19 WHO grading scale, and comorbidities, such as vascular, heart, lung, kidney, or neurologic diseases, malignancy, and metabolic syndrome) as well as laboratory values. All assessed variables are reported in Table 1.

**Table 1:** Overall clinical and laboratory characteristics of the study cohort.

feature	n and % of patients in which the feature was present	% of subjects in which the feature was not assessed
***cardiovascular disease***		
aortic stenosis	9 (3.8%)	32.3
atrial fibrillation	36 (15.4%)	32.6
carotid arterial disease	10 (4.3%)	32.3
chronic heart failure	21 (8.9%)	32.3
coronary heart disease	39 (16.7%)	32.9
hypertension	137 (58.1%)	32
myocardial infarction	20 (8.5%)	32.3
myocardial infarction or coronary heart disease	46 (19.7%)	32.9
peripheral vascular disease	12 (5.1%)	32.6
***delirium***	79 (22.8%)	0
***demographics***		
female sex	161 (46.4%)	0
age (years)	68.2 [55.5, 80.5]	0
***disease severity***		
ICU admission	81 (23.3%)	0
WHO grading scale		0
mild	143 (41.2%)	
moderate	135 (38.9%)	
severe	69 (19.9%)	
***kidney disease***		
kidney disease	26 (11.4%)	34
acute kidney injury	6 (2.6%)	34
chronic kidney injury	21 (8.9%)	32.3
***metabolic disease***		
diabetes with end-organ damage	6 (2.6%)	32.3
***malignancy***		
leukemia or lymphoma	8 (3.4%)	32.3
solid tumor	39 (16.6%)	32.3
tumor	47 (20.0%)	32.3
***neurological disease***		
epileptic history	21 (6.1%)	0
epileptogenic complications	7 (2.0%)	0
neurodegenerative history	47 (13.5%)	0
neuroimmunologic history	7 (2.0%)	0
neuroimmunologic complications	8 (2.3%)	0
neuropsychiatric history	25 (7.2%)	0
neurovascular complications	22 (6.3%)	0
neurovascular history	55 (15.9%)	0
***respiratory disease***		
asthma	8 (3.4%)	32.6
COPD and asthma	20 (8.5%)	32.6
COPD	12 (5.1%)	32.3
***outcome parameters***		
admission-discharge (days)	53 [14, 195]	62.8
death	58 (16.7%)	0
discharge destination		36.3
home	163 (73.8%)	
other hospital	13 (5.9%)	
nursing home	32 (14.5%)	
rehabilitation	13 (5.9%)	
***laboratory measurements***		
BUN (mg/dl)	51.5 [31.8, 101.5]	47
CRP (mg/dl)	12.3 [4.3, 20.9]	36.9
creatinine (mg/dl)	1.0 [0.8, 1.6]	37.2
ferritin (ng/ml)	89 [36; 167]	48.4
IL-6 (pg/ml)	45.6 [19.7, 189.5]	64.6
leukocyte count (1/μl)	8,870 [5,858, 13,068]	36.6
oxygen saturation (venous; %)	70 [45; 89]	48.4
pH (venous)	7.40 [7.38; 7.44]	58.5
pO2 (venous; mmHg)	36 [26; 46]	58.8
procalcitonin (ng/ml)	0.17 [0.07, 0.90]	43.5
sodium (mmol/L)	136 [133; 138]	37.2

The study cohort comprised 348 subjects. Numeric variables are reported as median and IQR. The numeric values reported for the laboratory measurements represent the most extreme value during the hospital stay (e.g., max. CRP, min. SO2). BUN: Blood urea nitrogen. COPD: Chronic obstructive pulmonary disease. CRP: C-reactive protein. IL-6: Interleukin-6.

The occurrence of delirium was significantly associated with the variables age (p = 0.01), prior history of neurodegenerative disease (p < 0.0001), COVID-19 disease severity as captured by the WHO grading scale (p < 0.0001) and the need for ICU admission (p < 0.0001), the inflammatory parameters leukocyte count, CRP, PCT and IL-6 (each p < 0.0001), and the renal function parameters BUN and creatinine (each p < 0.0001; all p-values corrected for multiple comparisons, see Table 2). All inflammatory parameters were consistently higher in subjects with delirium than those without delirium: the leukocyte count was almost twice as high (median values: 13,205/μl vs. 7,715/μl), CRP was twice as high (18.3 mg/dl vs. 8.9 mg/dl), PCT more than four times as high (0.53 ng/ml vs. 0.12 ng/ml) and IL-6 almost five times as high (127 pg/ml vs. 27 pg/ml). The renal function was worse in individuals with delirium, both in terms of serum creatinine (1.5 mg/dl vs. 0.9 mg/dl) and BUN (107 mg/dl vs. 42 mg/dl).

**Table 2:** Demographic, clinical and laboratory patient characteristics stratified by the occurrence of delirium.

	Without Delirium	With Delirium	adjusted p-values
n	268	79	
***cardiovascular disease***			
aortic stenosis	6 (3.3%)	3 (5.6%)	n. s.
atrial fibrillation	22 (12.2%)	14 (26.4%)	0.52
carotid arterial disease	9 (5.0%)	1 (1.9%)	n. s.
chronic heart failure	12 (6.6%)	9 (16.7%)	0.85
coronary heart disease	26 (14.5%)	13 (24.1%)	n. s.
myocardial infarction	13 (7.2%)	7 (13.0%)	n. s.
myocardial infarction or coronary heart disease	30 (16.8%)	16 (29.6%)	n. s.
hypertension	98 (54.1%)	39 (70.9%)	0.85
peripheral vascular disease	8 (4.4%)	4 (7.4%)	n. s.
***demographics***			
age (years)	65.7 [53.2, 79.3]	76.9 [61.4, 82.5]	0.01
female sex	133 (49.6%)	28 (35.4%)	0.85
***disease severity***			
ICU admission	36 (13.4%)	45 (57.0%)	<0.0001
WHO grading scale			<0.0001
mild	137 (51.1%)	6 (7.6%)	
moderate	103 (38.4%)	32 (40.5%)	
severe	28 (10.4%)	41 (51.9%)	
***kidney disease***			
kidney disease	19 (10.8%)	7 (13.2%)	n. s.
acute kidney injury	2 (1.1%)	4 (7.5%)	0.81
chronic kidney injury	17 (9.4%)	4 (7.4%)	n. s.
***metabolic disease***			
diabetes with end-organ damage	5 (2.8%)	1 (1.9%)	n. s.
***malignancy***			
leukemia or lymphoma	7 (3.9%)	1 (1.9%)	n. s.
solid tumor	31 (17.1%)	8 (14.8%)	n. s.
tumor	38 (21.0%)	9 (16.7%)	n. s.
***neurological disease***			
epileptogenic complications	4 (1.5%)	3 (3.8%)	n. s.
epileptogenic history	10 (3.7%)	11 (13.9%)	0.07
neurodegenerative history	25 (9.3%)	22 (27.8%)	<0.0001
neuroimmunologic complications	5 (1.9%)	3 (3.8%)	n. s.
neuroimmunologic history	5 (1.9%)	2 (2.5%)	n. s.
neuropsychiatric history	15 (5.6%)	10 (12.7%)	n. s.
neurovascular complications	15 (5.6%)	7 (8.9%)	n. s.
neurovascular history	33 (12.3%)	22 (27.8%)	0.05
***outcome parameters***			
admission-discharge (days)	41 [12, 194]	161 [21, 216]	0.97
death	37 (13.8%)	21 (26.6%)	0.32
discharge destination			<0.0001
home	149 (87.1%)	14 (28.0%)	
other hospital	7 (4.1%)	6 (12.0%)	
nursing home	10 (5.8%)	22 (44.0%)	
rehabilitation	5 (2.9%)	8 (16.0%)	
***respiratory disease***			
asthma	8 (4.4%)	0 (0.0%)	n. s.
COPD and asthma	18 (10.0%)	2 (3.7%)	n. s.
COPD	10 (5.5%)	2 (3.7%)	n. s.
***laboratory measurements***			
BUN (mg/dl)	42 [27; 71]	107 [52; 137]	<0.0001
creatinine (mg/dl)	0.9 [0.8; 1.3]	1.5 [0.9; 2.3]	<0.0001
CRP (mg/dl)	8.9 [3.0; 16.7]	18.3 [13.8; 26.6]	<0.0001
ferritin (ng/ml)	59 [28; 144]	157 [98; 416]	n. s.
IL-6 (pg/ml)	27 [14; 96]	127 [41; 275]	<0.0001
leukocyte count (1/μl)	7,715 [5,630; 10,855]	13,205 [9,272; 17,060]	<0.0001
oxygen saturation (venous; %)	73 [47; 90]	64 [41; 89]	n. s.
pH (venous)	7.41 [7.39; 7.45]	7.40 [7.36; 7.43]	n. s.
pO2 (venous; mmHg)	36 [28; 47]	36 [26; 43]	n. s.
procalcitonin (ng/ml)	0.12 [0.06; 0.41]	0.53 [0.17; 5.64]	<0.0001
sodium (mmol/L)	136 [133; 138]	135 [131; 137]	0.97

Numeric variables are reported as median and IQR. WHO = World Health Organization; COPD = chronic obstructive pulmonary disease; CRP = C-reactive protein; BUN = blood urea nitrogen; IL-6 = Interleukin-6; ICU = intensive care unit; POCT = point-of-care testing.

### Identification of important predictors for the occurrence of delirium

The strongest predictors for the occurrence of delirium in our COVID-19 cohort were, as reflected by the minimal depths (MD) values computed by the random-forest model (in descending order): blood urea nitrogen (MD: 3.33), age (MD: 3.75), disease severity (as captured by WHO grading, MD: 3.93), leukocyte count (MD: 4.22), the presence of a neurodegenerative history (4.43), ferritin levels (4.46), creatinine (MD: 4.59) and oxygen saturation (MD: 4.96) (Table 3).

**Table 3:** Strong predictors of the occurrence of delirium selected by a random-forest approach.

Variable	Minimal depth value
blood urea nitrogen	3.33
age	3.75
WHO grading scale	3.93
leukocytes	4.22
neurodegenerative history	4.43
ferritin	4.46
creatinine	4.59
pO2 (venous; mmHg)	4.96

The strength of variable for predicting the occurrence of delirium is reflected by its minimal depth value, with low minimal depth values indicating strong predictors.

### Effect of delirium on clinical outcome

While the occurrence of delirium was not significantly associated with hospital stay duration or increased mortality rate, it was associated with patient’s discharge destination: patients with delirium were less likely discharged home, but more often discharged to other hospitals, nursing homes or rehabilitation (Table 2). The occurrence of delirium was not associated with previously reported risk factors of a more severe disease course which might consecutively bear a higher risk for the occurrence of delirium, including male gender, presence of cardiovascular diseases or metabolic syndrome. Subject characteristics stratified by the presence of delirium and the respective adjusted p-values are reported in Table 2.

### Neuroradiological findings associated with delirium

In the subgroup of patients for whom neuroradiological parameters were available (n = 40, median age 70.5 years, range 40–90 years, 42% women, S1 Table for patient characteristics), we did not identify any factors associated with delirium (Table 4). Particularly, the occurrence of delirium was not associated with the presence of cerebral atrophy, the degree of microangiopathy or the presence of acute cerebrovascular events. The neuroradiological features stratified by the presence of delirium are reported in Table 4.

**Table 4:** Neuroradiological patient characteristics stratified by the presence of delirium.

	without delirium	with delirium	unadjusted p-values
n	27	13	
acute	3 (11.1%)	4 (30.8%)	0.19
chronic	7 (25.9%)	4 (30.8%)	n. s.
intracranial hemorrhage	3 (11.1%)	1 (7.7%)	n. s.
Fazekas score			0.33
0	11 (40.7%)	8 (61.5%)	
1	6 (22.2%)	4 (30.8%)	
2	4 (14.8%)	0 (0.0%)	
3	6 (22.2%)	1 (7.7%)	
global atrophy	15 (55.6%)	5 (38.5)	0.50
frontal atrophy	18 (66.7%)	6 (46.2%)	0.31
temporal atrophy	13 (48.1%)	3 (23.1%)	0.18
parietal atrophy	14 (51.9%)	5 (38.5%)	0.51

Neuroradiological findings were available in a sub-cohort of 40 subjects. Features of leukoencephalopathy were assessed according to the Fazekas score [22].

### COVID-19 related neuroimaging findings

Irrespective of the occurrence of delirium, presumably COVID-19 related findings were observed in 7/40 subjects (18%), including acute cerebral infarction (4/40, 10%), acute intracranial hemorrhage (3/40, 8%), with one hemorrhage resulting from a cerebral venous thrombosis, and hyperintensity of the olfactory nerve on T2 weighted MRI (2/40, 6%). We did not observe any (meningo-) encephalitic findings in our neuroradiologically examined patients. Further neuroradiological findings–presumably unrelated to COVID-19 –comprised chronic infarcts (9/40, 24%), microangiopathic white matter lesions (20/40, 50%) and other incidental findings, such as malignancies.

## Discussion

Our large single-center retrospective cohort study, conducted at a tertiary referral center, disclosed an incidence of 22.8% for the occurrence of delirium in hospitalized COVID-19 patients. As risk factors for delirium occurrence in COVID-19, our study identified (a) advanced age, (b) prior neurodegenerative disease, (c) COVID-19 disease severity (WHO grading, ICU admission), (d) markedly increased laboratory infection parameters, and (e) compromised renal function. Notably, the occurrence of delirium was not significantly associated with an increased in-hospital mortality of COVID-19 patients, but had a negative effect on patients’ discharge modality. Moreover, the occurrence of delirium was not associated with systematic differences in patients’ neuroradiological findings.

Our study comprised a large cohort of consecutive COVID-19 in-patients (n = 347), who were followed up throughout their entire hospital stay until discharge. All in-patients of our center were included, which allowed a representative assessment of the occurrence of delirium in the in-patient COVID-19 setting at our center. By contrast, previous studies have only assessed delirium in subgroups of COVID-19 patients (such as patients aged above 65 years [10] or intensive care patients [1,6,12]), only reported on shorter, and hence more selective observational periods [11], or only screened patients upon hospital admission, not following them up during the entire hospital stay [3].

We observed delirium in 22.8% across all hospitalized COVID-19 patients, and in 57.0% of the ICU-treated COVID-19 patients. Our study thus corroborates the results of previous studies that reported an overall incidence of delirium in 11%-33% of COVID-19 patients [9,11] and shows a higher incidence of delirium (more than 50%) for the intensive care setting [1,4,5,12,23]. Nevertheless, our study might even underestimate the incidence of delirium in the ICU, if one considers that some ICU patients were already transferred back to their referral hospitals before being weaned. This needs to be emphasized as other studies have described a higher predisposition to delirium in mechanically ventilated COVID-19 patients [24].

In a broader context, the overall incidence of delirium in our hospitalized COVID-19 patients was comparable to that of hospitalized *non-*COVID-19 patients, which has been reported to range from 6% to 56% [8]. Regarding the occurrence of delirium in ARDS due to other infectious causes, no significant differences have been reported between influenza patients (65.9%) and COVID-19 patients [25]. Overall, delirium appears to be a typical complication of acute stage coronavirus diseases, occurring not only in SARS-CoV-2, but also in other SARS coronavirus variants and MERS [26]. For patients with ARDS caused by *various* etiologies–not only viral respiratory infections–previous studies have disclosed an incidence of delirium comparable to that of patients with ARDS due to COVID-19 [27].

As risk factors for the occurrence of delirium in COVID-19 in the present study, we identified advanced age, a prior history of neurodegenerative disease, more severe course of COVID-19 (i.e., WHO categories moderate and severe, ICU admission), markedly increased laboratory infection parameters (such as leukocyte count and ferritin), and more severely compromised renal function. These factors identified by single-variable group comparisons were confirmed by a machine-learning approach (i.e., a random-forest classifier), which also allowed quantifying the relative importance of each variable for predicting the occurrence of delirium. Hereby, the variables BUN, age and disease severity (WHO grading) were demonstrated to be the strongest predictors of delirium. Our study thus, confirms and extends the major risk factors for delirium identified by previous studies [10]. Age in particular appears to constitute a significant predisposing factor to COVID-19-associated delirium and delirium has been described as an early and common manifestation of COVID-19 in older patients with a prior history of neurodegenerative disease [28]. The relevance of age as a predisposing factor for delirium in COVID-19 has also been underlined by another study, in which both patients’ age and the rate of delirium were higher than in our study [29]. Moreover, increased laboratory infection parameters (e.g., leukocyte count, CRP and ferritin) have been reported to be useful as predictive biomarkers for disease severity and overall COVID-19 prognosis [30,31]. Beyond inflammatory parameters, another objective biomarker for stratifying the risk of delirium in COVID-19 could be the blood level of neurofilament light, a biomarker that indicates ongoing neuronal damage [32].

Further factors previously reported to increase the likelihood of delirium in COVID-19, but not assessed in our study, comprise frailty, alcoholism, smoking, and polypharmacy [9,10,29]. For instance, it has been previously shown that delirium does not represent an independent predictor of mortality in frail patients with COVID-19 [33].

Our study succeeds in demonstrating a direct association of the occurrence of delirium with disease severity of COVID-19 as captured by the WHO disease grading scale, whereas other studies derived disease severity only indirectly from the necessity of intubation, the hospital stay duration and/or the presence of ARDS [1,10,12]. The risk factors age, clinical disease severity, increased laboratory infection parameters and increased renal retention parameters, which our study identified as predictors of COVID-19-associated delirium, have also been associated with the occurrence of delirium in otherwise critically ill patients in the absence of SARS-CoV-2 infection [8,34]. Consequently, one needs to bear in mind that delirium is prevalent across all patients admitted to ICU [27], including but not limited to those with COVID-19. For example, a study reported that–after adjustment for important covariates, such as acute and chronic health variables and analgetic/sedative medication–COVID-19 was no longer an independent risk factor for delirium in the ICU setting [35].

Unexpectedly, the occurrence of delirium in our COVID-19 cohort was not associated with certain factors previously reported to predispose to a more severe disease course–which might in turn result into a higher risk of delirium. In particular, delirium was not associated with male gender, cardiovascular disease, or metabolic syndrome–factors well associated with a severe disease COVID-19 course [36–38]. This observation is interesting as it suggests that the factors predisposing to a severe disease course of COVID-19 and the factors predisposing to COVID-19-associated delirium partially dissociate. Thus, further research is warranted to identify predisposing, but also preventive or protective factors for COVID-19-associated delirium.

In addition, we could not reproduce any significant associations between neuroradiological findings and the occurrence of delirium in our COVID-19 cohort as reported in other publications [39,40]. Remarkably, despite the association with prior neurodegenerative disease, the occurrence of delirium was not associated with cerebral atrophy–the neuroradiological manifestation of neurodegenerative processes and particularly dementia–or vascular brain damage, including microangiopathy and acute cerebrovascular events, which together could be expected to predispose to delirium.

Our study has several limitations: First, our dataset included several missing values, which were imputed–thus, our findings of the random-forest modelling need to be externally validated in the future. Second, it was a monocentric study conducted at a tertiary referral center, which might constrain the transferability of our findings to smaller hospitals or different settings. Third, we recommend validation of our retrospective findings in prospective studies. Fourth, our study population was recruited mainly during the first and second wave of COVID-19 in Germany, in which the wildtype variant (and to some degree the alpha variant) of SARS-CoV-2 was predominant [41], and before COVID-19 vaccination became available. Thus, we advise caution when generalizing the present findings to future pandemic waves which will likely be driven by other virus variants and modified by the effects of vaccination. Fifth, there might have been further biases, since certain laboratory measurements (e.g., IL-6, ferritin) may have been more often acquired in severely ill patients, and not evenly across our entire study cohort. Thus, patients with delirium may have been over-represented in these measurements as opposed to patients without delirium. Despite data imputation, we cannot exclude the possibility that some associations of certain variables with delirium might actually be less strong than reported. Sixth, as systematic information on the pre-morbid functional state (e.g., pre-morbid Barthel, PMRS)–going beyond the presence of the reported chronic conditions (e.g., neurodegenerative disease history)–was not available, we cannot exclude that the pre-morbid functional state might have confounded subjects’ outcome, as reflected for instance by their discharge destination. Finally, the missing association of COVID-19-associated delirium with neuroradiological findings needs to be considered preliminary, since the sample size of the neuroradiologically assessed sub-cohort was small, and neuroradiological scans were acquired as clinically indicated, but not in a consecutive manner.

Our study observed a negative effect of the occurrence of delirium on patients’ discharge modality, hereby confirming previous studies. Yet, we did not observe significant associations with the length of hospital stay or mortality, as reported in previous research [3,11–13]. This finding might be partially explained by the specialized expertise of our center in the management of ARDS and the fact that, up to the start of our recruitment period, relevant experience in the management of COVID-19 had already been acquired during the first wave of the pandemic.

In conclusion, the risk of delirium in COVID-19 can be stratified based on the factors advanced age, prior neurodegenerative history, disease severity (as assessed by the WHO grading scale, ICU admission and oxygen demand), high infection parameters, and increased renal retention parameters, thus allowing early identification of patients at risk of developing COVID-19-associated delirium. With specialized management at experienced centers, the high morbidity and mortality of COVID-19-associated delirium may be drastically attenuated.

## Supporting information

S1 Table Patient characteristics (n = 40) of the subgroup with neuroradiological parameters. (DOCX)
