# Supplementary material for: Delirium in hospitalized COVID-19 patients: Predictors and implications for patient outcome
Source: PLoS One. 2022 Dec 22;17(12):e0278214. doi: 10.1371/journal.pone.0278214 (PMC9778494; doi:10.1371/journal.pone.0278214)
Supplement: S1 Table — (DOCX) [file pone.0278214.s001.docx]

***Supporting Information***

| **variable** | **n = 40 (%)** |
| --- | --- |
| delirium | 13 (32.5%) |
| acute cerebral infarction | 7 (17.5%) |
| chronic cerebral infarction | 11 (27.5%) |
| intracranial hemorrhage | 4 (10.0%) |
| **Fazekas score** |  |
| 0 | 19 (47.5%) |
| 1 | 10 (25.0%) |
| 2 | 4 (10.0%) |
| 3 | 7 (17.5%) |
| global atrophy | 20 (50.0%) |
| frontal atrophy | 24 (60.0%) |
| temporal atrophy | 16 (40.0%) |
| parietal atrophy | 19 (47.5%) |

**Table S1.:** Patient characteristics (n=40) of the subgroup with radiologic parameters
